# Detecting Changes and Avoiding Catastrophic Forgetting in Dynamic Partially Observable Environments

**DOI:** 10.3389/fnbot.2020.578675

**Published:** 2020-12-23

**Authors:** Jeffery Dick, Pawel Ladosz, Eseoghene Ben-Iwhiwhu, Hideyasu Shimadzu, Peter Kinnell, Praveen K. Pilly, Soheil Kolouri, Andrea Soltoggio

**Affiliations:** ^1^Department of Computer Science, Loughborough University, Loughborough, United Kingdom; ^2^Mathematical Sciences, Loughborough University, Loughborough, United Kingdom; ^3^Teikyo University Graduate School of Public Health, Tokyo, Japan; ^4^School of Mechanical, Electrical and Manufacturing Engineering, Loughborough University, Loughborough, United Kingdom; ^5^HRL Laboratories, Malibu, CA, United States

**Keywords:** POMDP, PSR, continual learning, catastrophic forgetting, lifelong learning, neural network

## Abstract

The ability of an agent to detect changes in an environment is key to successful adaptation. This ability involves at least two phases: learning a model of an environment, and detecting that a change is likely to have occurred when this model is no longer accurate. This task is particularly challenging in partially observable environments, such as those modeled with partially observable Markov decision processes (POMDPs). Some predictive learners are able to infer the state from observations and thus perform better with partial observability. Predictive state representations (PSRs) and neural networks are two such tools that can be trained to predict the probabilities of future observations. However, most such existing methods focus primarily on static problems in which only one environment is learned. In this paper, we propose an algorithm that uses statistical tests to estimate the probability of different predictive models to fit the current environment. We exploit the underlying probability distributions of predictive models to provide a fast and explainable method to assess and justify the model's beliefs about the current environment. Crucially, by doing so, the method can label incoming data as fitting different models, and thus can continuously train separate models in different environments. This new method is shown to prevent catastrophic forgetting when new environments, or tasks, are encountered. The method can also be of use when AI-informed decisions require justifications because its beliefs are based on statistical evidence from observations. We empirically demonstrate the benefit of the novel method with simulations in a set of POMDP environments.

## 1. Introduction

A useful skill for an agent that explores the world and learns to act in it is the ability to predict what happens next (Geisser, 1993). One way is to try to learn a model of the world so that predictions are generated within the agent and compared with observations to improve the model. However, this idea assumes that it is possible to learn a large and static model of the entire world. In reality, it is more feasible to try to learn a model of a subset of the world, i.e., an environment. Therefore, an agent may interact in different environments at different points in time. This is a condition that challenges learning algorithms that often need to be set manually by a user that labels tasks or substitutes models for each new task or environment.

A more effective agent would be able to learn different tasks or environments more autonomously, incorporating new knowledge without forgetting the skills learned in a previous task or environment. To accomplish this, an agent needs to learn an environment and detect when a change occurs, or when a completely new environment is met. By doing so, a lifelong learning agent will also remember previously learned environments, and quickly recover their models if old conditions return.

The majority of current machine learning approaches, however, assume that learning occurs in one static environment. These approaches make it possible to optimize policies for one problem, but do not scale well to learning multiple problems, or to learning in an incremental way more tasks over a lifetime (Thrun, 1998). Approaches known as meta reinforcement learning (Finn et al., 2017; Rothfuss et al., 2018; Rakelly et al., 2019; Zintgraf et al., 2019) are designed to learn multiple tasks, but they assume that a signal is given when the task changes. While this is a step toward learning more tasks sequentially, such algorithms still require a teaching signal that labels different tasks. Additionally, reinforcement learning algorithms are intended to optimize a policy that maximizes reward (Sutton and Barto, 2018). Therefore, the knowledge of the environment is implicit in the policy and shaped by the reward function.

One method to quickly adapt to changes in the environment is provided by reinforcement learning approaches that explicitly model the environment, and therefore can rapidly search the parameter space when those change. These approaches, known as model-based reinforcement learning (Doya et al., 2002; Nagabandi et al., 2018; Lecarpentier and Rachelson, 2019), require the model to be hand-designed, and also learn policies with the aim to maximize a reward. In short, because reinforcement learning aims to provide actions that maximize reward, their applicability is limited when there are no actions or rewards available.

If an environment does not provide rewards, it can still be explored and learned with the aim, e.g., to predict future events. The concepts of Markov chain and of Markov decision process (MDP) are abstractions to model an environment when actions are, respectively, absent or present (Bellman, 1957). An extension of MDPs are partially observable Markov decision processes (POMDPs) that account for the fact that observations from a system do not always reveal the state. POMDPs have many important applications in the real world, e.g., the airplane collision avoidance system ACAS X is based on POMDP models (Kochenderfer et al., 2015). POMDPs have also been used to model brain information processing for decision making under uncertainty (Rao, 2010), and to model working memory in the brain (Todd et al., 2009). While POMDPs are a flexible tool to model a variety of real world systems, the assumption of partial observability of the underlying states in the observed environment is precisely what makes it difficult to derive accurate POMDPs from data.

Because POMDPs can include a reward function, much of the research in learning POMDPs falls under reinforcement learning theory and is intended to find an optimal policy in a rewarding environment (see e.g., Shani et al., 2005). One exception is the Baum-Welch algorithm (Rabiner, 1989), designed to generate Hidden Markov Models, that can be adapted for POMDP environments by incorporating actions. One limitation of this method is that it requires knowing the number of states in advance, and works best when provided with an initial estimate of the underlying POMDP.

Predictive state representation (PSR) is a general representation of a POMDP that does not need to learn the underlying states. PSRs, instead, learn the probabilities of future observations. Due to the nature of PSR methods, which learn directly from observations rather than trying to find hidden underlying states, discovering and learning an accurate PSR of a POMDP environment is faster than trying to reconstruct the underlying MDP (Littman et al., 2002). A variety of algorithms have been proposed to improve the learning of PSRs such as transformed predictive state representations (TPSRs) (Rosencrantz et al., 2004) and compressed predictive state representations (CPSRs) (Hamilton et al., 2013). Some algorithms improve the learning method, often utilizing TPSRs and CPSRs (McCracken and Bowling, 2005; Yun-Long and Ren-Hou, 2009; Liu et al., 2016; Downey et al., 2017), while others allow the agent to learn in more complex domains, e.g., with continuous action or observation spaces (Wingate and Singh, 2007; Boots et al., 2013).

Other parametric models such as neural networks (Bishop, 1995) can also take a history of recent actions and observations as input, and be trained to predict the next observation. These approaches are less explainable, but have grown in popularity with the resurgence of neural networks, the use of deep and recurrent networks, and powerful hardware for training (Schmidhuber, 2015).

The approaches cited so far assume that the environment is stationary. Therefore, we can hypothesize that under dynamic conditions where the agent switches between environments over time, such approaches will either learn an average of the environments, or learn accurately the most recent environment while forgetting the previous ones. One example of dynamic conditions is an autonomous driving problem in which a vehicle encounters different environments, such as different driving rules or weather conditions. In theory, two or more POMDP models that alternate over time can be modeled as one single POMDP in which a non-observable state determines the sub-part of the model that generates the current data. However, this approach is likely to increase the complexity of the model significantly. Thus, a hierarchical approach in which different POMDP models are used to predict different environments may be more scalable.

Assume, e.g., a system in which a transition from a state A to B occurs consistently with probability 1, but after some time has passed, the environment dynamics change such that state A leads to C with probability 1. The challenge in learning this case with one single model is that rather than capturing the hidden state, the model could learn that the environment transitions from A to B or to C with a probability of 0.5 for each state. This is true on average, but inaccurate at any particular point in time. As a consequence, the result will be either a model with low accuracy, if a slow learning rate is used, or catastrophic forgetting if a faster learning rate is used.

The idea presented in this paper is to explicitly learn such hierarchically nested hidden states by means of a statistical framework that selects different predictive models to fit a data stream at different times. The proposed approach tracks the probability of a current window of data of fitting different models, and thus the probability of an agent being in one of many possible environments when the only cues, e.g., observations and actions, are implicit in the data stream. This is done by comparing the expected frequencies of observations derived from predictive models with the observed frequencies in the current data stream. Discrete observations and actions follow multinomial distributions, thus, performing χ^2^ tests is a viable method of estimating the probability of the new observed data to fit a learned predictive model.

An important consequence of assessing a model's probability of fitting the current data is that data points at different times can be assigned to different models to improve them separately and independently. By doing so, we can learn different models under the assumption of stationary conditions and implement continual learning of multiple environments, thus avoiding catastrophic forgetting in dynamic POMDPs. The proposed method provides an evidence-based and explainable algorithm to justify the belief of the system. Moreover, the novel approach does not need reward signals to learn models for different environments, making it a more general method than reward-based approaches such as reinforcement learning.

The next section provides the background on POMDPs that is necessary to introduce the novel algorithm that we name adaptive model detection (AMD). We also briefly introduce PSRs and a simple neural network as baseline model learners to be employed by the novel AMD algorithm explained in section 3. Simulation results are presented in section 4 followed by a discussion and conclusion.

## 2. Background

Predictive models such as predictive state representations (Littman et al., 2002), neural networks and POMDPs have been extensively used in the past to model dynamical systems with discrete representations. This section provides the background for these approaches that lay the framework for the method in this paper.

### 2.1. Predictive State Representation (PSR) and POMDP

Predictive state representation (PSR) is a model to predict observations in stochastic environments, including POMDPs. A POMDP is defined as a hextuple {S,A,T,O,Ω,R}, where S is the set of underlying MDP states; A is the set of actions; *T* is the transition function, T:A×S×S→[0,1], which gives the probability of transitioning from one state to another given the action taken; *R* is the reward function; O is the set of observations; and Ω is the set of conditional observation probabilities. POMDPs differ from MDPs in that the current observation is not sufficient for an agent to be able to determine its underlying state.

Let *t* be a finite stream of action-observation pairs. Then *t* is a test, and we let T be the set of all tests. Let the history *h*_*i*_ ∈ *H* of the agent at time *i* be the stream of action-observation pairs $a_{j}\in A,o_{j}\in O,\forall j\in \left[0,i\right)\cap \mathbb{N}$ observed up to time *i*.

A PSR (Littman et al., 2002) of an environment consists of a set of core tests, *Q*; a set of |*Q*|-dimensional *m*_*a,o,t*_ vectors for all a∈A,o∈O,t∈Q; and an initial state.

The set of core tests, *Q*, is a finite subset of T, with the property that *P*(*t* ∣ *h*) for all t∈T,h∈H can be found as some *linear combination* of the probabilities *P*(*q* ∣ *h*) for all *q* ∈ *Q*. The empty test, ϵ = {}, is always included in *Q*, such that *P*(ϵ ∣ *h*) = 1 for all *h* which are possible under the PSR model. The PSR state vector after observing history *h*, *y*(*h*), is a (1 × |*Q*|) vector which holds *P*(*q* ∣ *h*) for each *q* ∈ *Q*. By stacking the rows of *m*_*a,o,t*_ for all *t* ∈ *Q*, we obtain a (|*Q*| × |*Q*|) *projection vector* *M*_*a,o*_ for every length 1 test (*a,o*). For all *h* ∈ *H* we have that $P\left(o|h,a\right)=y\left(h\right)\times M_{a,o}^{T}$. Projection vectors for longer tests can be created by multiplying projection vectors for shorter tests. For example, *M*_*a*_1_,*o*_1_,*a*_2_,*o*_2__ = *M*_*a*_2_,*o*_2__ × *M*_*a*_1_,*o*_1__, where × is matrix multiplication.

To maintain an accurate state vector, *m*_*a,o,t*_ must be available for all a∈A,o∈O,t∈Q. Let *Q* = {*t*_1_, *t*_2_, …, *t*_*n*_}. This can be used to obtain the state vector at time *i*, *y*(*h*_*i*_), given the *a*_*i*_, *o*_*i*_ action observation pair observed at timestep *i*, and *y*(*h*_*i*−1_). Recall that the PSR state vector contains the probabilities of each core test, and let *y*_*j*_(*h*) denote the element in *y*(*h*) corresponding to core test *t*_*j*_. Then, the probability of each core test can be found as follows. For all *t*_*j*_ ∈ *Q*:

(1) $$\begin{array}{r} y_{j}\left(h_{i}\right)=P\left(t_{j}|h_{i-1},a_{i},o_{i}\right)=\frac{y\left(h_{i-1}\right)\times m_{a_{i},o_{i},t_{j}}^{T}}{y\left(h_{i-1}\right)\times m_{a_{i},o_{i}}^{T}}\;. \end{array}$$

Equivalently, we can use the previously defined projection vectors:

(2) $$\begin{array}{r} y\left(h_{i}\right)=\frac{y\left(h_{i-1}\right)\times M_{a_{i},o_{i}}^{T}}{y\left(h_{i-1}\right)\times m_{a_{i},o_{i}}^{T}}\;. \end{array}$$

From the definition above, it follows that PSRs can give an indication of the probability of certain transitions to occur. In particular, the following theorem specifies the probability of observing particular action observation pairs:

**THEOREM 1**. Given that the state vector is *y*(*h*_*i*−1_) at time *i* − 1, the probability of seeing observation *o*_*i*_ after taking action *a*_*i*_ at time step *i* is given by the following equation

(3) $$\begin{array}{r} P\left(o_{i}|h_{i-1},a_{i}\right)=y\left(h_{i-1}\right)\times m_{a_{i},o_{i}}^{T}\;. \end{array}$$

Proof: By construction. Note that the state vector contains enough information to accurately predict future observations even in partially observable environments. The state vector acts not only as a prediction, but also as an internal state for the PSR model.

A natural question to ask is, ‘what is the predictive state of an empty history *y*(ϵ)?’. If the environment is known to always start in a given underlying state, the corresponding predictive state may be used. However, this is a strong assumption in general. If we assume the agent is likely to start in each underlying state proportionally to the amount of time previously spent in that state, *y*(ϵ) can be set to the *stationary distribution*. The stationary distribution, which is the weighted expected value of *y* over all time steps, is given by

(4) $$\begin{array}{r} y\left(\epsilon \right)=\frac{\sum_{h\in H}y\left(h\right)}{\left|H\right|} \end{array}$$

where *H* is the set of all histories. As *H* is an infinite set, this is calculated instead from the histories that the agent has seen. When calculated this way, the stationary distribution may change depending on the policy followed by the agent. Additionally, as the stationary distribution represents a distribution over all states, it may not be a state that the agent can reach through normal exploration; however, it represents a positive probability for all states previously visited.

The state space of the PSR is the set of states that can be generated recursively by $y=\frac{y^{\prime }\times M_{a,o}^{T}}{y^{\prime }\times m_{a,o}^{T}}$, for all a∈A,o∈O, where *y*′ is known to be in the state space of the PSR, and $y\times m_{a,o}^{T}$ is non-zero. In order to generate the set of states, an initial state *y*′ must be assumed to be in the state space. Sometimes an initial state is provided when the starting state of the environment is known. However, when no initial state is provided, the stationary distribution may be used.

There are several algorithms for learning PSRs offline, but relatively few for learning and discovering tests online. One such algorithm is the constrained gradient algorithm (McCracken and Bowling, 2005), which we use in our experiments for learning PSRs.

### 2.2. Neural Network Predictors

A simple neural network (Bishop, 1995) can be trained to predict observations in a given environment. Given a time window of duration *i*, a neural network can take the observations o∈O and the actions a∈A and predict the new observation *o* at time *i* + 1. If the softmax transfer function is used to produce output probabilities, the network can also be trained to predict the probability of each observation at *i* + 1 in stochastic environments. The difference between the prediction and the observation can be used to train such a system with gradient descent. As for the PSR model explained in the previous section, a neural network predictor can be trained effectively only if stationary conditions are assumed during the training phase. Thus, changes in the environment such as those occurring in dynamic POMDPs (see next section) are challenging conditions that this study addresses.

Neural networks, PSRs, and other predictive models have one thing in common: they give as output a prediction of the probability of seeing each possible observation next. The sum of probabilities of seeing each observation is 1. Let *K* be a predictive model, O be the set of possible observations, and A be the set of possible actions. Then, we define *P*(*o* ∣ *K*_*i*_) to be the predicted probability of observing *o* at time *i* given by *K*. Additionally, we define $P\left(O|K_{i}\right)$ as the probability distribution over all observations at time *i* given by *K*.

### 2.3. Dynamic POMDPs

Assume that an environment remains stationary for a certain amount of time. Under this assumption, it is possible to learn a model of the environment (e.g., a PSR, as detailed in McCracken and Bowling, 2005). If, after a certain amount of time, the environment changes, the continual training of the same model will lead to inaccurate predictions at first, and catastrophic forgetting of the first environment in the long term. This is because the assumption of stationary conditions is not valid, and one model tries to learn an average distribution of two or more distributions that occur in different environments. These changing environments are similar to switching hidden Markov models (SHMMs) investigated in Chuk et al. (2020) and Höffken et al. (2009).

Let 𝕍 = {*V*_1_, *V*_2_, …, *V*_*m*_} be a set of distinct POMDP environments. A dynamic POMDP environment, *D*, behaves as a single environment *V*_*i*_ ∈ 𝕍 for a number of time steps, *n*_0_, before changing its behavior to another environment, *V*_*j*_ ∈ 𝕍. The dynamic environment *D* may switch to any environment in 𝕍 every *n*_*k*_ time steps, with *k* ∈ ℕ and *n*_*k*_ ≫ 1. Effectively, these dynamics can be seen as hierarchical large stationary models where a state variable *z* ∈ ℕ determines the specific environment *V*_*z*_ at a given time. However, *z* is not observable and can only be derived inferring which specific environment from the set 𝕍 matches the current stream of data. We assume that transitions between environments in 𝕍 occur with considerably lower frequency than state transitions within the environments *V*_*z*_. This assumption reflects two points: (1) in real world scenarios, generally, two environments can be thought of as being distinct when transitions from one to another occur rarely, otherwise it makes more sense to consider them as one environment; (2) for a model to learn and predict one environment, it is necessary to experience the environment for a minimum number of steps that capture transitions within it.

Such dynamic conditions occur typically when an agent learns to predict an environment, e.g., to navigate in a house, and is then required to learn a new somewhat different environment, e.g., to navigate in an office. The new environment might bear a similarity with the previous one, but also significant differences. Desirable skills of an agent include the ability to detect that there is a new environment, the ability to learn the new environment quickly, possibly exploiting previous knowledge, and also retain the knowledge of the previous environment (avoiding catastrophic forgetting). The aim of this study is precisely to achieve such capabilities as explained in the next section.

## 3. Adaptive Model Detection

The idea and the method for detecting different environments and training different models according to such detection is explained in this section. We name our new approach adaptive model detection (AMD) because it detects likely models to fit the data, and works with adaptive models that evolve as new data is collected.

### 3.1. Statistical Model Selection

Given a set of statistical models that each predict an environment, a question that can be asked is: what model is best at predicting a given stream of data? Several approaches have been proposed in the literature to perform *model selection* (Cox, 2006). Approaches for model selections are based on the information theory such as the Akaike information criterion (Akaike, 1974) and the Bayesian (or Schwarz) information criterion (Schwarz, 1978). The idea is to measure the information that is lost due to the difference in statistical distributions between the model and the data using the Kullback-Leibler divergence (Kullback, 1997). By doing so, it is possible to select a best fitting model that minimizes this difference. Different statistical methods, however, may be more or less appropriate or accurate according to a number of factors including the number of data points and the assumptions on the statistical distributions that are being observed.

Given a recent window of data from one environment, *hypothesis testing* can be used to accept or reject the null hypothesis that the distribution of the environment data matches the distribution of the model-generated data (Lehmann and Romano, 2006). The ability to accept or reject such a null hypothesis is a valuable tool, particularly in the context of explainable AI applications in which a model is used to predict a data stream. If the current data stream has a very low *p*-value, it is reasonable to reject the null hypothesis that the model is correct. On the contrary, if the null hypothesis cannot be rejected, the model offers a good approximation and it is therefore reasonable to (1) consider it as a good-enough predictor and (2) use new data to further improve it via a training process.

We assume that (1) a data stream is generated by one environment *V*_*i*_ from the set 𝕍; (2) that we want to learn and identify which model *K*_*i*_ ∈ 𝕂 describes the current data stream, including if none of the current models describe the data; (3) the data stream produces a set of finite discrete or categorical outcomes. Therefore, we use hypothesis testing instead of model selection, so that we can determine when none of the existing models fit the data, allowing us to create a new model. To identify which specific *V*_*i*_ is generating the data, the problem can be formulated as selecting the corresponding model *K*_*i*_ that maximizes the likelihood

(5) $$\begin{array}{r} \underset{i}{\mathrm{argmax}}\hat{L}=P\left(h|K_{i}\right) \end{array}$$

where *h* is a limited time window of recent input data.

It is important to note that there are no guarantees that a current set of models, 𝕂, can accurately predict a corresponding set of environments 𝕍. However, the key idea tested is: assuming we can select the most fitting model given by Equation (5), then we can associate a particular time window of the data stream and use it to improve the model *K*_*i*_ to maximize $\hat{L}$. Therefore, such an approach can be used both to learn and to find the best set of models that fit a set of environments.

The data generated by the set of actions and observations in a POMDP as described in the previous section form a multinomial distribution. Thus, the χ^2^ test can be used to accept or reject the null hypothesis that a recent time window of data is generated by a given model. In the next sections, the procedure to compute the degrees of freedom and the χ^2^ *p*-values is presented.

### 3.2. Calculating Degrees of Freedom

The degrees of freedom (DF) of a statistical model *K* corresponds to the number of independent parameters in the model, and is required to perform statistical tests such as χ^2^. Predictive models have in common the ability to predict the probability distribution of the next observation given a history or internal state. Note that we are not trying to find the number of independent parameters in the predictive agent, but in the underlying model the agent predicts. Let $o^{\prime }\in O$. If, for all $o\in \left\{O\backslash o^{\prime }\right\}$ we know the value of *P*(*o* ∣ *K*_*i*_), then

(6) $$\begin{array}{r} P\left(o^{\prime }|K_{i}\right)=1-\underset{o\in \left\{O\backslash o^{\prime }\right\}}{\sum }P\left(o|K_{i}\right)\;. \end{array}$$

Therefore, the number of independent parameters for each prediction is |O|-1 as the final observation's probability can be inferred from the others. Each prediction may be independent in the predictive model, thus, we assume that the predictive model is an approximation to a statistical model (the underlying POMDP) with a number of independent states that is much smaller than the length of a history of data points. To estimate the number of independent states in the underlying model, we cluster together similar predictions in the predictive model. These can be clustered according to the predictive model's hidden states, the prediction of the next observation, or the prediction of several next observations.

### 3.3. Sampling and Clustering Probabilities

The adaptive model detection algorithm (AMD) keeps a history window *W* of up to *L* recent prediction observation pairs, where *L* is a parameter of the algorithm. The choice of *L* affects the behavior of the algorithm as shown in the results section with an analysis of different values for *L*.

Knowing how many times each prediction has been made is necessary to determine whether the environment behaves as the predictive model expects. To count the number of times different predictions are given by a learning model, it is necessary to cluster such predictions that are expressed as vectors with possibly slightly different probabilities values. Let $C_{K}$ be the set of all clusters made by AMD for the predictive model. For a given cluster $c\in C_{K}$, let $\bar{c}$ be the mean of the distributions $P\left(O|K_{i}\right)\in c$ in the cluster, and $\bar{c}\left(o\right)$ therefore be the mean probability of observing observation *o*.

AMD uses the DBSCAN clustering algorithm (Ester et al., 1996) to form clusters of predictions given by a model. The scikit-learn (Pedregosa et al., 2011) vanilla implementation of the algorithm is used. As the data changes over time, the DBSCAN algorithm may form clusters differently on each timestep. This does not pose an issue, as $\bar{c}$ can be recalculated at each timestep, meaning that even when the clusters change, the expected and observed frequencies will be similar in an accurate agent. DBSCAN does not include into clusters those outlier points which seem to not fit into larger clusters. Such a property is advantageous in our context because: (1) the mean distribution of a cluster $\bar{c}$ is therefore not affected by an outlier that was forced into it, and (2) clusters must be formed of a certain minimum size, the advantage of which is explained in section 3.5.

### 3.4. Calculating χ^2^ *p*-Value

To compute the χ^2^, AMD counts as *X*_*c,o*_ the number of times in the history that each observation *o* follows predictions in each cluster *c* for all *c* ∈ *C* and o∈O. The number of times each observation *o* is expected to follow predictions in a given cluster is given by $E_{c,o}=|c|\times \bar{c}\left(o\right)$.

Thus, from Pearson (1900),

(7) $$\begin{array}{r} \chi^{2}=\underset{c\in C}{\sum }\underset{o\in O}{\sum }\frac{X_{c,o}-E_{c,o}}{E_{c,o}}\;. \end{array}$$

Effectively, χ^2^ measures how well the actual data matches the expected data, with higher values meaning that the observed data does not match the expected data well. For a general model, some values of *X*_*c,o*_ and *E*_*c,o*_ may be equal or close to 0, corresponding to impossible (or never observed) transitions. For these cases when *X*_*c,o*_ = *E*_*c,o*_ = 0, the value $\frac{X_{c,o}-E_{c,o}}{E_{c,o}}$ is set to 0.

Knowing the χ^2^ value and DF allows us to find the *p*-value, which represents the probability of the observed data coming from the expected data distribution. This function is available in most statistical programs, code libraries, and toolkits as part of the χ^2^ implementation^1^.

### 3.5. χ^2^ Testing for Adaptive Model Detection

Following the guidelines in (Yates et al., 1999), a χ^2^ test is considered to be sufficiently accurate when “no more than 20% of the expected counts are less than five, and all individual expected counts are one or greater.” Accordingly, a minimum history length is necessary to be able to perform the test, and the longer the history, the more accurate the test is expected to be. Unfortunately, with an arbitrary large POMDP, even a long history does not guarantee that all possible prediction observation pairs have been seen. Additionally, while a long history length makes *p*-values more accurate, a long history means that the assessment of the probability for a model can be done only over a long period of time, potentially losing granularity on the precise point of the transition.

Even with a long history length, if the agent encounters a sequence of observations it does not expect, it may produce an internal state or prediction unlike any it has generated before. Due to this, the cluster containing the unusual state or prediction could be small enough that the expected number of times a given observation follows states in the cluster is lower than 1. Conveniently, DBSCAN has a minimum cluster size, so such small clusters can be avoided entirely, unless of course the probability of observing a particular observation from states in a cluster are very low.

An AMD that tests data against multiple predictive models *K*_1_, *K*_2_, …, *K*_*n*_ keeps track of the *p*-value of each one, and uses this tracked *p*-value to determine which environment it is most likely to be in. This allows AMD to select which model is trained at any point in time in a statistically motivated way, contributing to explainable decisions in AI. Additionally, detecting which environments are likely at given points in time opens up the possibility of applying a different policy based on the current environment.

Note that as long as the *p*-value is above the threshold at which the null hypothesis is rejected, it does not matter whether the value is low, high, or fluctuating. The AMD algorithm is summarized with pseudo code in Algorithm 1.

## 4. Simulation Results

The effectiveness of the algorithm in a variety of settings is tested with computer simulations. The set of environments is introduced in section 4.1. The effect of the history length parameter *L* on the detection speed and stability is investigated in section 4.2. In section 4.3, the algorithm is extended to demonstrate how labeling incoming data can be used to continually train separate models, and thus avoid catastrophic forgetting.

### 4.1. Chosen Environments

**Figure 1:**
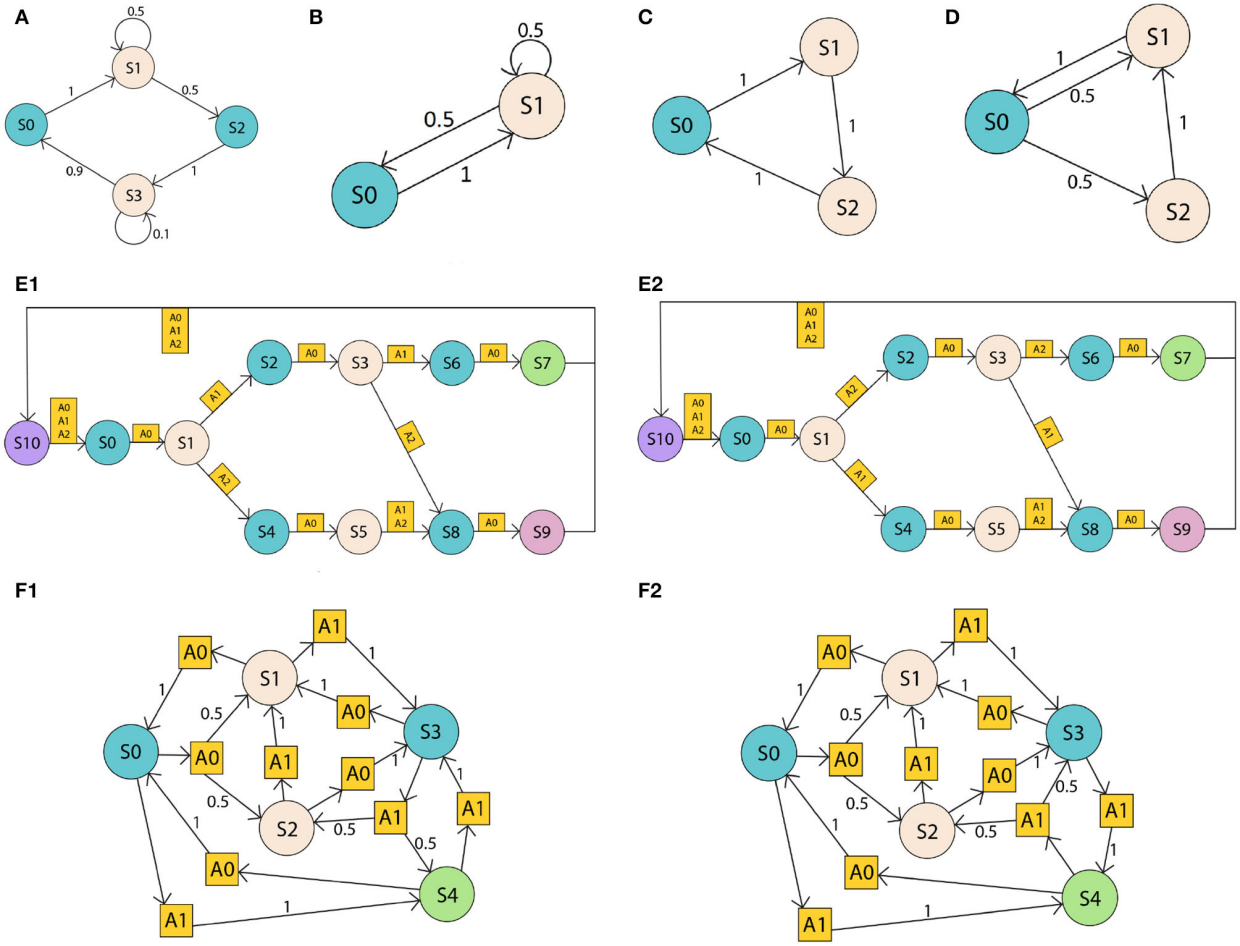
Set of POMDP environments. States are represented with circles, actions with squares, and transition probabilities with numbers on arrows. The color of the state corresponds to the observation. Partial observability derives from the fact that different states provide the same observation. The first set **(A–D)** are simple Markov chains where transitions occurs without actions and probabilities indicated near the transition arrows. The second set **(E1,E1)** are more complex problems where actions determines different paths in a cycle but have deterministic transitions. Environments **(F1,F2)** are POMDPs with some deterministic and some stochastic transitions.

The proposed algorithm was tested on a set of POMDPs of various size and complexity. The first set of problems (Figures 1A–D) is a series of uncontrolled POMDP environments, i.e., Markov chain environments, with only 2 observations (as there are more states than observations, the observation is given by the color of the state in Figure 1). These environments appear simple at first, however, they have different states and transition probabilities, and, due to their stochastic nature, some environments could be mistaken for others based on the data generated by exploration. For example, environment C creates a data stream that can be produced by environment D. However, when interacting with the environment over a longer period, the observations reveal the data stream is more likely to be generated by environment C than environment D.

The next set of environments, Figures 1E1,E2, represents a decision process originating in S0 where one sequence of actions takes the agent to S7, and all other sequences lead to S9. The challenge in this set is that the observations do not reveal the distance from S0, and thus make it hard for an agent to locate itself along the graph as it progresses from left to right. E1 and E2 have two further variations, E3 and E4 (not shown). E3 is the same as E1 but the transitions from S1 have inverted actions. Similarly, E4 is the same as E2 but transitions from S1 have inverted actions. These four environments are very similar in structure, but they require different policies to be traversed from *S*0 to *S*7.

Finally, environments Figures 1F1,F2 have most of their transition probabilities being exactly the same. This means that transitions in the data stream distinguishing the two environments occur less frequently than in some other environments.

### 4.2. Speed and Reliability of Detections: Impact of History Length

**Figure 2:**
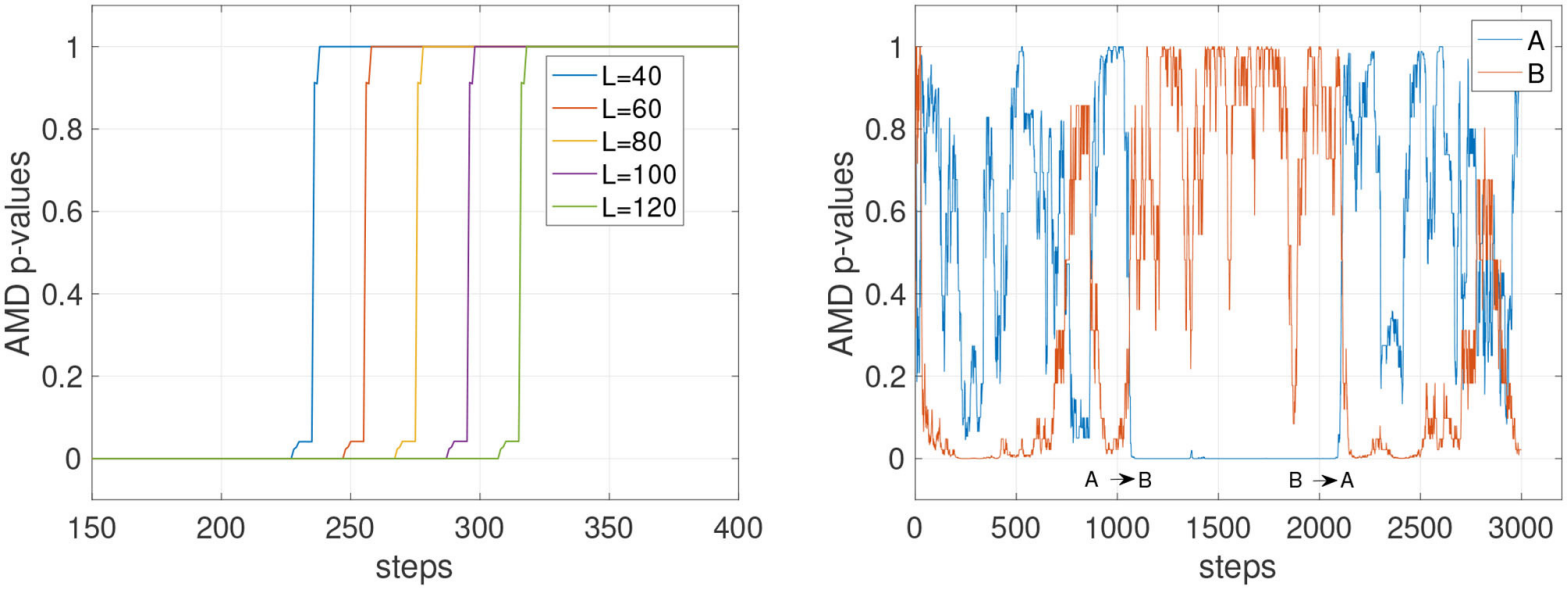
AMD *p*-values with deterministic and stochastic environments. **(Left)** Tracking of environment C with different history lengths (parameter *L*). C is deterministic and the *p*-values are consistent at 0 and 1, but the time taken to detect the change in environment varies with the history length. **(Right)** AMD *p*-values for the model A and B with the stochastic environments A and B and *L* = 120. Tracking stochastic environments results in *p*-values predicting the wrong environment more frequently.

The experiments in this section assess overall stability of the detection and the impact of the history length expressed by the parameter *L*. Figure 2 shows the effect of different history lengths when tracking the probability of an accurate PSR model for environment C. In Figure 2 (left), the values of *L* for 40, 60, 80, 100, and 120 are shown. In all cases, the AMD shows consistent *p*-values of 0 and 1, indicating that the model can confidently determine which model the data stream belongs to. The longer the history, the slower the change in *p*-value, confirming the intuitive notion that longer histories require more time steps to reveal a change in the environment. When assessed on the stochastic environments A and B (Figure 2, right), the dropping *p*-values indicate that stochasticity is a significant confounding factor in the detection of the environment.

**Figure 3:**
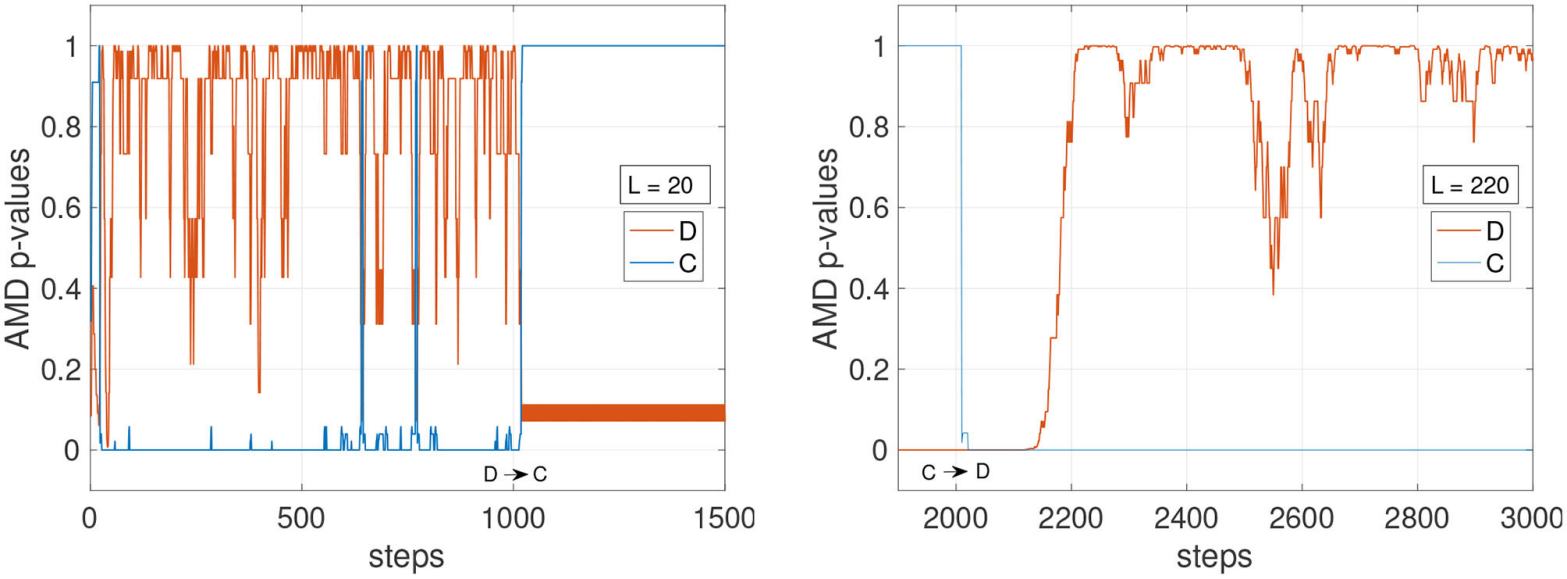
AMD *p*-values for the models of environments C and D. **(Left)** With a short history length (*L* = 20), the *p*-values for the environment D occasionally indicate the data matches environment C, and not environment D. **(Right)** With a longer history length (*L* = 220), the *p*-value for model D becomes more stable, not dropping below 0.3.

It can be concluded that a small *L* is advantageous to detect changes more readily only if the environment is predominantly deterministic. When the environment has stochastic transitions, a longer history might be necessary to guarantee the stability of the detection. To further assess these dynamics, Figure 3 shows the comparison of a short and a long history window (*L* = 20 and *L* = 120) on the deterministic environment C and the stochastic environment D. The AMD *p*-values show that while C can be tracked reliably with both *L* = 20 and *L* = 120, environment D (orange line) causes the *p*-value to oscillate, although with less amplitude, even with *L* = 120.

**Figure 4:**
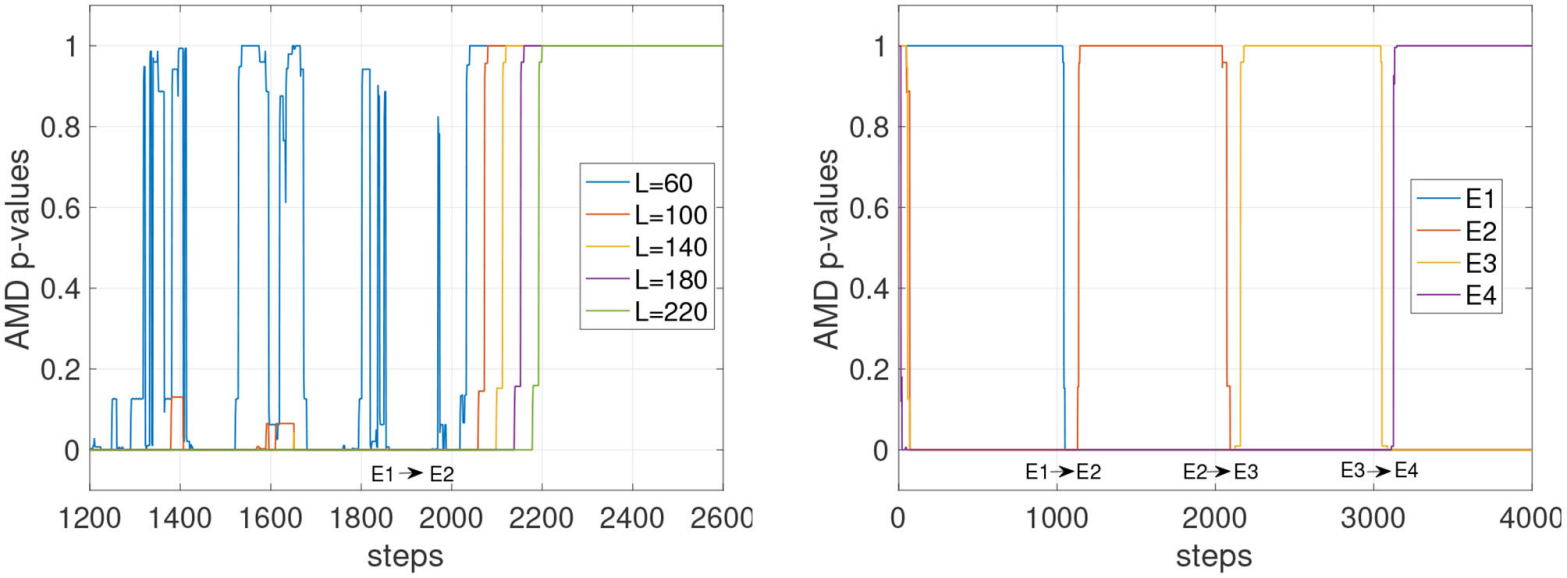
Stability of detections for the deterministic set of models E. The agents traversing this environment use a random policy, where actions are chosen from the set of possible actions which can be taken at each state. **(Left)** The environment transitions from E1 to E2. The graph shows *p*-values for the environment E2. **(Right)** *p*-values for all four models are shown while all four environments alternate (*L* = 220).

**Figure 5:**
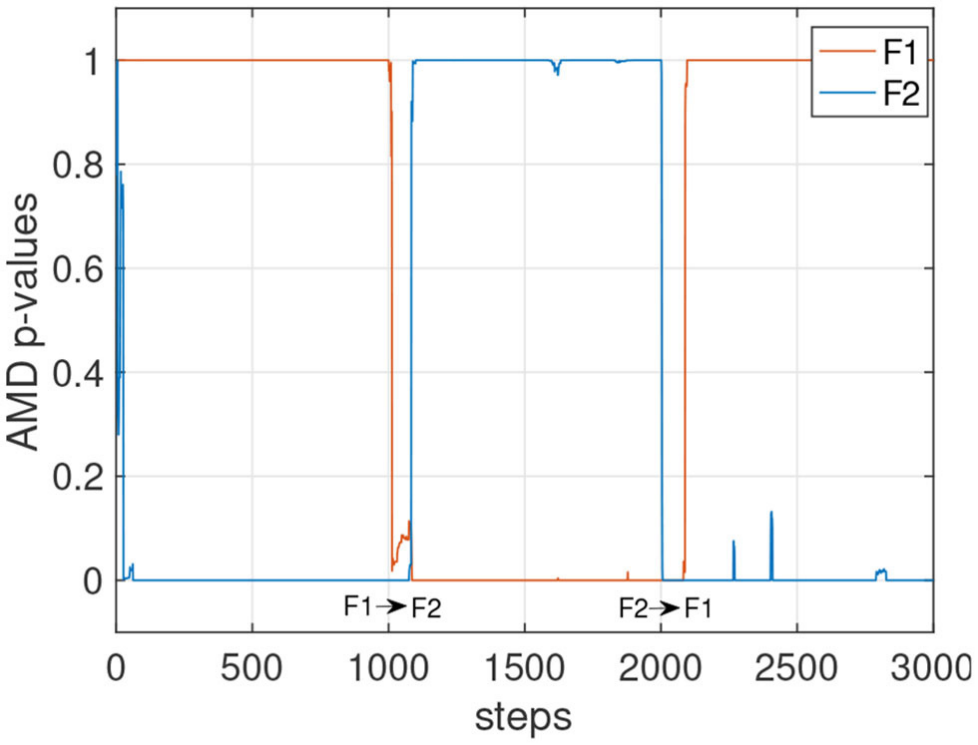
*p*-values for the models of the set F with *L* = 120. Agents traversing this environment utilize a random policy.

Other factors that can affect the stability of the *p*-values could include the complexity and the similarity with other environments. In Figure 4 (left), the tracking of the environments E1 and E2 is shown with *L* varying from 60 to 220. The faster settings (*L* = 60 and *L* = 100) appear to detect E2 when still in E1. With *L* = 140, the detection becomes more reliable, and with *L* = 180 and *L* = 220 the detection is accurate, although slower after step 2,000 to detect the transition from E1 to E2. Figure 4 (right) shows the *p*-values for all four E models tracked simultaneously. Despite the similarity of these four environments, the *p*-values show high confidence in determining which model currently matches the data stream. A similar result is also observed in Figure 5 where the environments of the set F are tested. The stochasticity in this set does not affect significantly the stability of the *p*-values. This is because the environment is primarily deterministic, and although the points where the data streams differ do not occur often, the *p*-value drops significantly when they do occur.

### 4.3. Avoiding Catastrophic Forgetting With Continual Learning of Multiple Models

The ability to detect which environment the current stream of data belongs to allows the system to train different models independently, and thus implement continual learning and avoid catastrophic forgetting. The scenario devised in this section is when two unknown environments X and Y alternate while a learning system is trying to learn them from the data stream. This condition is particularly challenging because the data stream is generated by two different environments, both unknown. Therefore, there is an obvious bootstrap problem. How can the AMD know when the environment changes before any environment has been learned?

A reasonable assumption to overcome this problem is to assume that the data stream is initially generated by a single environment for a certain amount of time, so that one single model can be at least partially trained on the initial data stream.

#### 4.3.1. AMD With Constrained Gradient PSRs

**Figure 6:**
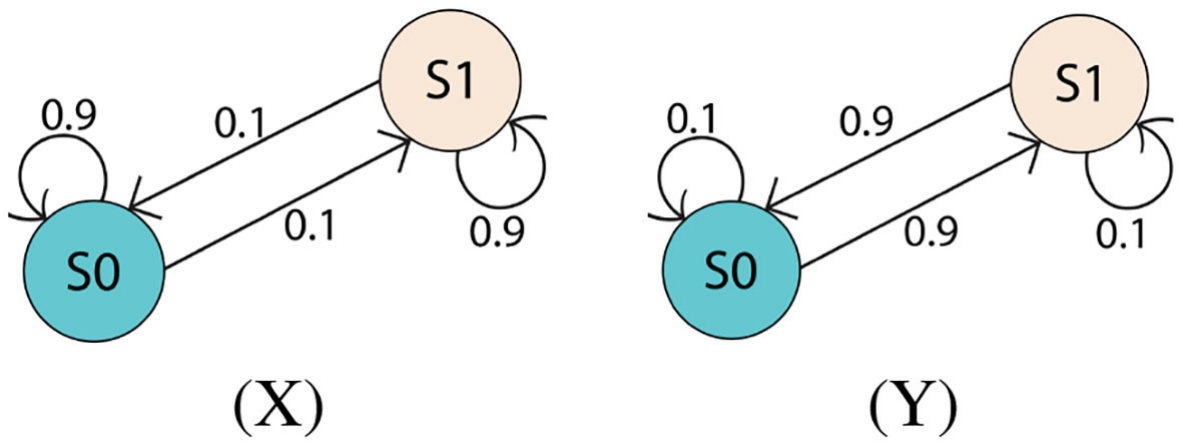
Environments X and Y. These environments have only two states, but are stochastic. Environment X is more likely to remain in its current state at each time step, whereas environment Y is more likely to transition between states.

**Figure 7:**
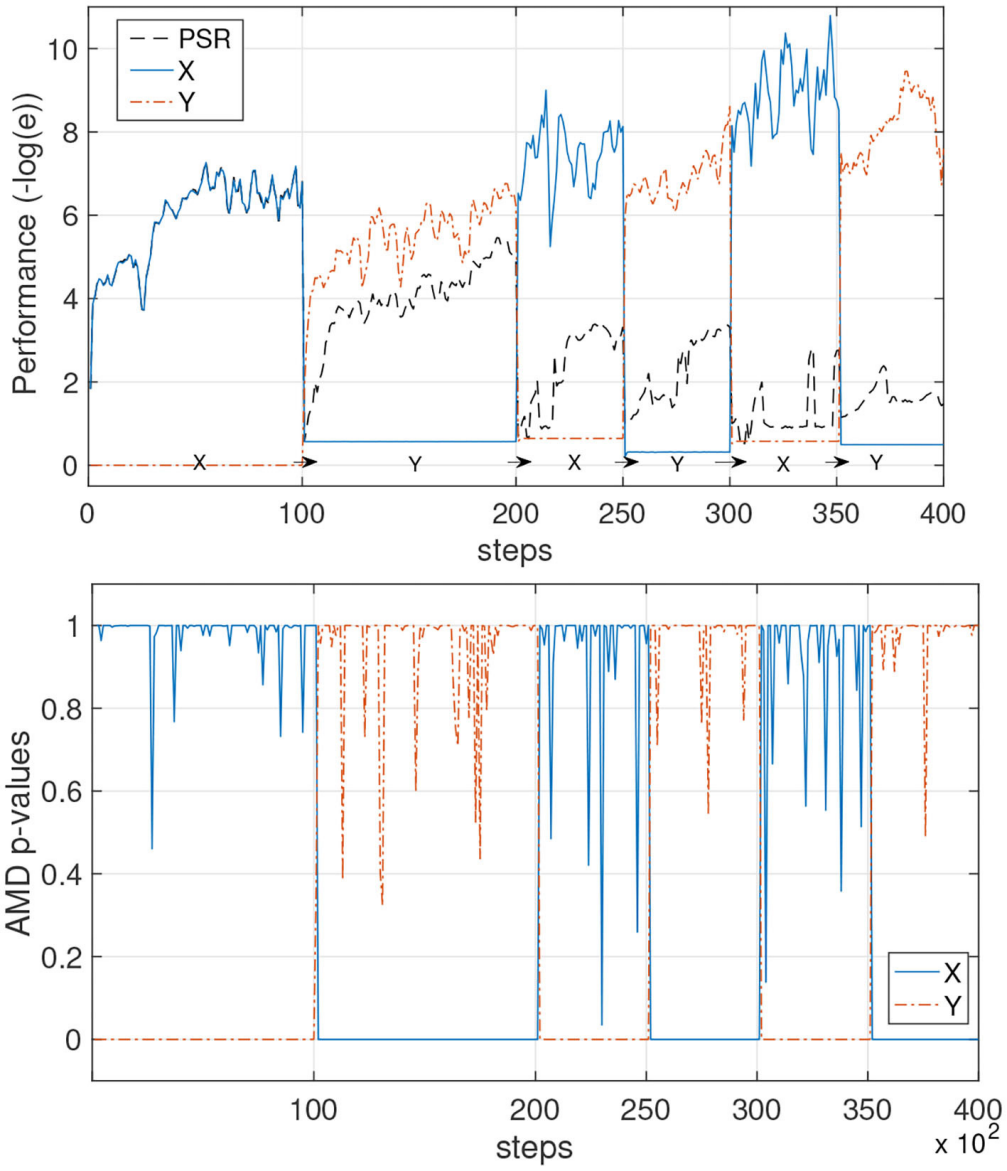
Avoiding catastrophic forgetting with AMD on PSR learners. The unknown environments X and Y alternate overtime. **(Top)** The performance is measured as the negative log of the prediction error of each model. A single PSR (black line) is trained on the data stream and it learns an average of the two environments. The azure continuous line and the orange dash line show the AMD-guided learning: first X is learned, and when the data switches to environment Y, a new model Y is also learned. Subsequently, data points originating from the two environments are used to further improve each model separately. **(Bottom)** The AMD *p*-values for the AMD-guided learners are shown during the process.

Two simple but highly stochastic environments, shown in Figure 6, are chosen for this test as depicted in Figure 7. The learning setup for this first test uses the constrained gradient PSR learner. It starts to learn a first model while environment X produces data for the first 10k steps. When the *p*-value suddenly drops and remains low at step 10,000, the AMD clearly indicates that the first model is not valid anymore. Thus, the new data is used to train a new model. A similar process occurs for the following environment changes: the PSR with the highest *p*-value is trained, and the other is left idle. The AMD continues to track the probabilities of each model. Effectively, each chunk of data that is identified by the AMD as belonging to one model is used to train that model only and thus enables continual learning and prevents catastrophic forgetting.

As a baseline, we ran the constrained gradient PSR learning algorithm with the same parameters, but without AMD detecting switches in the environment. The agent learns well until time step 10,000. At time step 10,000–20,000, the agent also learns the second environment well, although it is not able to reach the same performance as the PSR with AMD. From time step 20,000 onwards, it is clear that the agent has experienced catastrophic forgetting, as each time the environment is switched, the performance decreases dramatically.

In these experiments, the error is calculated as the average prediction error (the difference between the predicted probabilities of the next observation and the actual probabilities of the next observation) over 10,000 time steps in an independent data stream.

#### 4.3.2. AMD With Neural Network Learners

**Figure 8:**
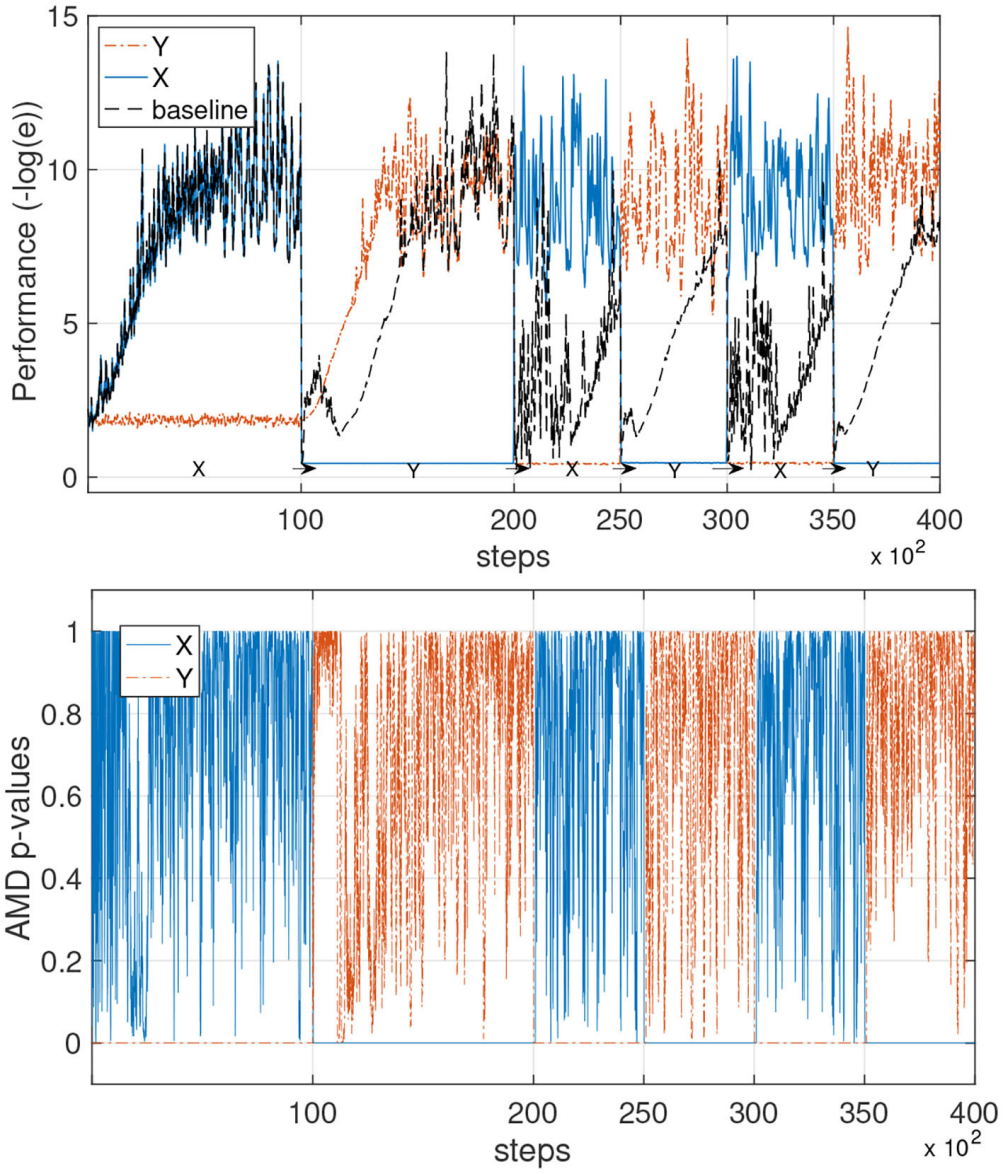
Avoiding catastrophic forgetting with AMD on neural networks models. This experiment is the same as that in the previous figure, but uses neural network learners instead of the constrained gradient algorithm. **(Top)** The performance of the AMD-enabled models is shown in the environments X and Y. The baseline is capable of continuous learning, but forgets the previous environment each time there is a switch. **(Bottom)** The AMD *p*-values for the AMD-guided learner are shown during the process.

To validate the AMD with the neural network learning models, we employed a simple three-layer network whose details are specified in the Appendix 6.1.2. Figure 8 shows the performance and *p*-values when the AMD is applied in combination with the neural network models.

We observe the same learning dynamics that were obtained with the PSR learner, although the neural network learner appears to achieve slightly better performance. The single model (baseline) shows the typical learning curves when different tasks or environments are learned sequentially, with catastrophic forgetting occurring each time the environment switches. The AMD instead can accurately determine when the environment switches and use the data to train two different models.

## 5. Discussion

The results presented in the previous section show that the idea to use statistical tests to determine the best fitting model to label data is a promising venue of research. Various aspects of the algorithm and of the experimental results prompt interesting questions.

The first important aspect that was investigated in section 4.2, the impact of history length, shows that the readiness in detection and stability are opposed objectives that need to be balanced. However, stochasticity appears to be the main factor that requires longer histories to guarantee stability. We speculate that, while high levels of stochasticity are an obvious obstacle to learning, it would be possible to learn also such features of the environments and incorporate them in future developments of the AMD. One possibility is to introduce an adaptive history length that could reduce if an environment is predominantly deterministic, and increase in length when highly stochastic transitions require more data points to acquire meaningful statistics.

A second observation is that the dynamic properties of a series of POMDPs can include both sudden changes and slow progressive drifts. In the case of drifts that progressively increase the distance between the distributions of the model and the environment, there will be a race between the adaptation speed of a learning algorithm and the AMD *p*-values. If the learning algorithm is fast enough to track the drift, the AMD will maintain a high *p*-value, thus maintaining confidence in the current model. This condition, however, would lead to progressive forgetting of the original distribution. If, on the other hand, the drift in the environment is faster than the speed at which the learning algorithm can adapt, the AMD will see the corresponding *p*-value drop and either select a different model, or create a new model to learn the new data distribution. While we did not investigate these conditions, the problem of deciding whether an environment is drifting to a new distribution, or changing significantly to warrant the instantiation of a new model, is a relevant aspect of lifelong learning worth of future studies.

The AMD is intended as a framework that is independent of the specific learning algorithm used to learn a model. However, it is worth pointing out that the AMD is limited by the underlying learning model. In fact, while the *p*-values of sub-optimal models could be low, and thus lead to model rejection or further learning, there are cases in which this is not true, leading to simpler models having higher *p*-values than more accurate ones. In fact, a *p*-value could be high when the environment is more complex than the model. Consider, e.g., an environment that generates a data stream of observations 0, 0, 1, 1, 0, 0, 1, 1, …, where each observation 1 or 0 occurs twice in a row. A model that predicts that after each 1, the next observation will be 0 or 1 with equal probability will score a high *p*-value although a better model could be learned. In short, the AMD *p*-values might not always provide the best metrics to assess the quality of a model. While choosing the simplest model to fit the data might prove effective to prevent overfitting and agrees with the Occam's razor principle, further analysis might reveal how best to integrate the AMD algorithm with specific learning methods.

Given a set of models, one interesting question is what approach is more effective when a new model is necessary to learn a new environment. In the context of lifelong learning, a desirable property is that of exploiting previous knowledge to accelerate the learning of new tasks. It is possible that the AMD could facilitate such a forward transfer by instantiating a new model that matches some properties of the new data. While this problem was not touched in this study, the AMD may provide useful statistical insights to inform the creation of new models.

The set of problems proposed in this study appears simple at first. However, it is worth noting that partial observability and stochasticity make it difficult to derive the correct model from observations even in relatively simple environments. Additionally, the complexity of an environment might derive from a large input space, e.g., when using raw images in a navigation task. We speculate that the use of the AMD in combination with large neural models for feature extraction could allow the extension of this method to more complex problems.

Finally, it is important to note that this study introduces the idea of model selection from statistics in the domain of dynamic POMDPs without rewards. We could not identify existing methods that could be used for a direct performance comparison. However, with the addition of a reward function, this study could be extended to incorporate a policy component, and thus place the approach in the field of reinforcement learning. Given the large amount of research in reinforcement learning, this extension would open several exciting research directions and comparisons with recent RL and meta-RL approaches.

## 6. Conclusion

This study introduces an algorithm that aims to address a limitation of many current learning systems: the inability to monitor a non-stationary data stream while learning from it. The proposed system, named adaptive model detection (AMD), monitors the data stream generated by partially observable Markov decision processes with the aim to assess the probability of the data fitting a given model. Statistical tests determine (1) whether the null hypothesis that a current model produces the data can be accepted or rejected and (2) which specific model from a set is more accurate to predict a recent window of data. The novel algorithm was tested with two types of predictive models, PSRs and neural networks. The simulations show that the approach is not only useful for quickly adapting to changes in an environment, but can also be useful to associate a stream of data to a particular environment. By doing so, it is possible to continuously train different models for different environments, and thus prevent catastrophic forgetting while learning multiple environments. The approach can be extended to address a wide set of problems beyond the limited scope of the environments tested here. The method could be valuable in AI applications where critical decisions require an evidence-based and justifiable process. When multiple environments are presented sequentially and require incremental learning without labels, rewards, or signals that a change has occurred, the approach presented can be used to implement continuous lifelong learning abilities.

## Data Availability Statement

Publicly available data were analyzed in this study. This data can be found here: https://github.com/JupiLogy/adaptive-model-detection.

## Author Contributions

JD developed the novel algorithm, wrote the computer code and performed the experiments. JD and AS devised the research plan and methods, analyzed the results, plotted the graphs and wrote the paper. PL and EB-I performed and analyzed experimental results. PP, SK, and PK contributed to the formulation of the research hypotheses. HS and SK provided support for the statistical method. All authors contributed to writing the final manuscript.

## Conflict of Interest

SK and PP were employed by HRL Laboratories. The remaining authors declare that the research was conducted in the absence of any commercial or financial relationships that could be construed as a potential conflict of interest.

## Footnotes

^1^In our implementations, the scipy function scipy.special.chdtrc is used.
