# Supplementary material for: Detecting Changes and Avoiding Catastrophic Forgetting in Dynamic Partially Observable Environments
Source: Front Neurorobot. 2020 Dec 23;14:578675. doi: 10.3389/fnbot.2020.578675 (PMC7787001; doi:10.3389/fnbot.2020.578675)
Supplement: Supplementary file 1 [file Presentation_1.pdf]

APPENDIX

1 IMPLEMENTATION WITH DIFFERENT PREDICTIVE MODELS

1.1 Predictive State Representation

The online constrained gradient algorithm is used to learn the different environments. The set of core tests is given in advance, with discovery turned off. The AMD algorithm is unaffected by discovery, so if the set of core tests is not known in advance, discovery can be turned on in constrained gradient's learning.

Our implementation sets the initial learning rate of the constrained gradient algorithm to 0.5, with the learning rate decaying by 10% every 2,000 time steps. The constrained gradient algorithm uses a history length of 200, whilst AMD uses $L = 40$.

The learning and predicting parts of the constrained gradient algorithm were separated. As the stored state history *is* the knowledge of the constrained gradient agent, the stored history is not updated when a prediction is made, only when the agent is asked to learn.

If the agent is asked not to learn for multiple consecutive time steps, then continuing the agent's training from its most recent state in history negatively affects the agent's performance if that state is not accurate for the timestep when learning is resumed. For this reason, we introduced a parameter to determine whether or not the agent trained on the most recent time step. If the agent did not train on the last time step but is asked to on the next, the agent removes states from history until the latest state in history matches with the current predicted state. This way the agent continues learning with a minimised effect on performance.

For the constrained gradient agent, the AMD does not cluster predictions based on the prediction of the next time step, but on the PSR state vector, which consists of predictions spanning multiple time steps in the future. This allows the clustering to be more accurate, especially in the case where two states may have the same probability of the next observation, but not observations further in the future. If only the prediction of the next observation is used to cluster, in this case, two separate states in the underlying environment would be grouped into a single cluster in AMD.

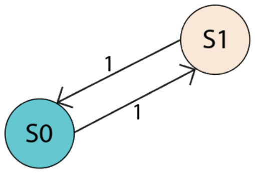

Figure 1. Example of a simple MDP to illustrate modifications to the PSR algorithm that are necessary when impossible observations are observed.

When an observation with a predicted probability of 0 is observed, it is a clear indication that the current model is not valid. Due to the PSR formulation presented in section 2.1, the PSR state vector becomes inaccurate. To illustrate the process with a practical example, consider the simple environment shown in Fig. 1. A possible PSR for this environment has the core tests $\{\epsilon, \text{'blue'}\}$. In this case, the stationary distribution is $[1, 0.5]$, the PSR state vector for state $S0$ is $[1, 0]$, and

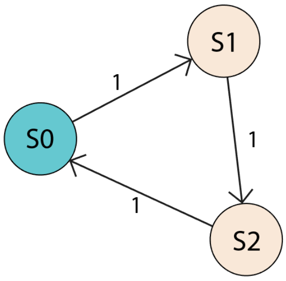

Figure 2. An environment where multiple states have the same prediction probabilities for the next observation.

the PSR state vector for state $S1$ is $[1, 1]$.

$$m_\epsilon = [1, 0], \quad m_{\text{'blue'}} = [0, 1], \quad m_{\text{'cream'}} = [1, -1].$$

If the current PSR state vector at time i is $y(h_i) = [1, 0]$ (corresponding to state $S0$), and observation 'blue' occurs at time $i + 1$, the new state vector will not be possible to calculate due to division by zero. This condition can be see in the operation to find the element in $y(h_{i+1})$ corresponding to the empty test $y_\epsilon(h_{i+1})$:

$$y_\epsilon(h_{i+1}) = y_\epsilon(h_i, \text{'blue'}) = \frac{y(h_i) \times m^T_{\text{'blue'}}}{y(h_i) \times m^T_{\text{'blue'}}} = \frac{[1, 0] \times [0, 1]^T}{[1, 0] \times [0, 1]^T} = 0/0$$

This case can be prevented by setting the state vector as the default state $y(\epsilon)$ whenever $y(h) \times m^T_\epsilon = 0$. Whenever $P(\epsilon \mid h_i) \neq 0$, $y(h_i) = \frac{y(h_{i-1}) \times M_{a_i,o_i}}{y(h_{i-1}) \times m_{a_i,o_i}}$.

Although the constrained gradient algorithm was used, AMD can be used with any PSR learner.

1.2 Neural Networks

When training, the neural network is fed the 5 most recent actions and observations as input. The network is trained based on prediction loss from the next observation given by the environment. The error - which is not fed to the agent, only used to measure performance - is calculated by comparing the agent's prediction of the next observation with the environment's true probability. Three layers were used: the input layer of size 10; a hidden layer of size 10; and an output layer of size 2, according to the possible number of observations in the environment. Softmax is used on the output layer.

AMD assumes the network's predictions are accurate for the environment it is supposed to have learned. The clusters are formed based on the output of the neural network, which is the agent's prediction of the probability distribution of the next observation. Therefore, the state and prediction provided to AMD are the same.

Note that the neural network has some potential issues which should be considered when working with more complex environments. Any length of input is not long enough to produce an accurate output if the environment is an infinite Markov model. LSTMs may be a better choice. We used a vanilla neural network as we only intend to show the value of AMD, rather than the performance of the network. Additionally, in POMDPs where some different underlying states

share the same probability distribution of the next observation (for example, in Fig. 2, both S_0 and S_1 have the same probability of observing the “cream” observation next), the neural network’s output does not distinguish between the underlying states. Using a hidden layer to form clusters instead may help distinguish between such states at the cost of increasing computational time.

2 PSEUDO-CODE

Algorithm 1 describes the AMD algorithm for detecting the probability of a predictive model’s current observations coming from the environment in which it was trained [1].

Algorithm 1: Probability tracking of predictive models

```
Input: hist_max          // desired maximum length of history kept
Set S to empty list          // history of states and predictions
Set hist to empty list          // window of history
Set C as empty set          // for storing clusters and cluster labels
Set act and exp as 0 for each cluster observation pair
while True do
    Input: a, o          // next action observation pair
    Input: s, P          // agent's next state and prediction
    Append [s, P] to S
    Append [a, o] to hist
    // remove old states, decrease act and exp values
    if len(hist)> hist_max then
        Remove S[1] from S
        Remove hist[1] from hist
        C ← DBSCAN(states in S)       // recalculate clusters
        Recalculate exp and act
    end
    // get probability of observed data matching environment
    Calculate degrees of freedom as DF
    p ← p-value given by χ^2
    // with actual values act, expected values exp, and DF degrees of freedom.
end
```

[1] The full implementation can be found at https://github.com/JupiLogy/adaptive-model-detection/ .
